# Huangkui lianchang decoction attenuates experimental colitis by inhibiting the NF-κB pathway and autophagy

**DOI:** 10.3389/fphar.2022.951558

**Published:** 2022-08-22

**Authors:** Xudong Cheng, Jun Du, Qing Zhou, Bensheng Wu, Haodong Wang, Zhizhong Xu, Shuguang Zhen, Jieyu Jiang, Xiaopeng Wang, Zongqi He

**Affiliations:** ^1^ Suzhou TCM Hospital Affiliated to Nanjing University of Chinese Medicine, Suzhou, China; ^2^ Affiliated Hospital of Nanjing University of Chinese Medicine, Nanjing, China; ^3^ Jingren Medical Laboratory, Nanjing, China; ^4^ Suzhou Foreign Language School, Suzhou, China

**Keywords:** ulcerative colitis, Huangkui lianchang decoction, mesalazine enema, NF-κB pathway, autophagy

## Abstract

Ulcerative colitis (UC) is a chronic inflammatory colorectal disease characterized by excessive mucosal immune response activation and dysfunction of autophagy in intestinal epithelial cells. Traditional herbal preparations, including the Huangkui lianchang decoction (HLD), are effective in UC clinical treatment in East Asia, but the underlying mechanism is unclear. This study evaluated the therapeutic effects and associated molecular mechanisms of HLD in UC *in vivo* and *in vitro*. A C57BL/6 UC mouse model was established using 2.5% dextran sulfate sodium. The effects of HLD on the colonic structure and inflammation in mice were evaluated using mesalazine as the control. The anti-inflammatory effects of HLD were assessed using disease activity index (DAI) scores, histological scores, enzyme-linked immunosorbent assay, immunohistochemistry, immunofluorescence, and western blotting. HLD displayed a protective effect in UC mice by reducing the DAI and colonic histological scores, as well as levels of inflammatory cytokines and NF-κB p65 in colonic tissues. NCM460 lipopolysaccharide-induced cells were administered drug serum-containing HLD (HLD-DS) to evaluate the protective effect against UC and the effect on autophagy. HLD-DS exhibited anti-inflammatory effects in NCM460 cells by reducing the levels of inflammatory cytokines and increasing interleukin 10 levels. HLD-DS reduced p-NF-κB p65, LC3II/I, and Beclin 1 expression, which suggested that HLD alleviated colitis by inhibiting the NF-κB pathway and autophagy. However, there was no crosstalk between the NF-κB pathway and autophagy. These findings confirmed that HLD was an effective herbal preparation for the treatment of UC.

## GRAPHICAL ABSTRACT

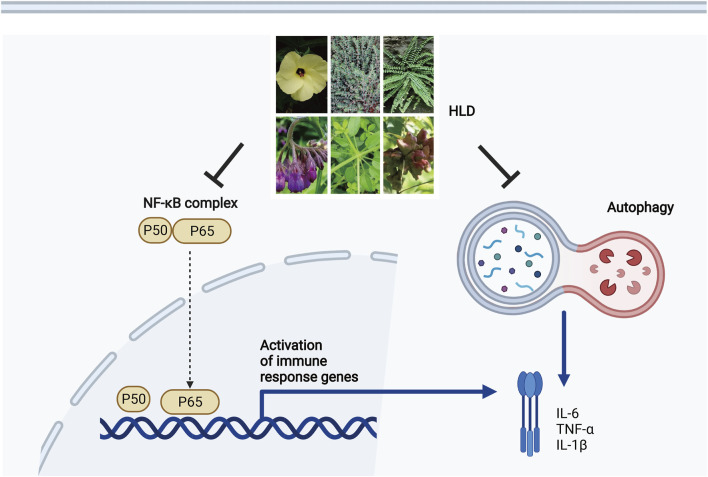

## Introduction

Ulcerative colitis (UC) is a chronic, recurrent, inflammatory intestinal disease of unknown etiology with a prolonged and recurrent course and can involve any colon part (Raine et al., 2022). Recently, UC incidence has been on the rise worldwide, seriously endangering the lives and health of people and increasing medical expenses (Wei et al., 2021).

The primary goal of UC treatment is to prevent disability and avoid colectomy and colorectal cancer by maintaining remission (Turner et al., 2021; Raine et al., 2022). Currently, the primary medicines used to treat UC include corticosteroids, 5-aminosalicylic acid, immunosuppressants, and biologics. Although these drugs have reduced the UC recurrence rate, they generally have disadvantages such as side effects, high price, reduced quality of life and patient satisfaction, and increased medical burden with long-term use (Raine et al., 2022). Therefore, it is necessary to find an alternative treatment that is safe, effective, and well-tolerated. Studies have found that a variety of herbs have excellent anti-inflammatory activities and can be used to alleviate intestinal inflammation.

Autophagy is essential for maintaining intestinal mucosal immunity, intestinal epithelial barrier integrity, and microbial defenses (Wu et al., 2019). Autophagy dysfunction can disrupt the intestinal mucosal barrier and consequently lead to the development of inflammatory bowel disease (IBD) (Lassen and Xavier, 2018; Trentesaux et al., 2020). Therefore, targeting autophagy to repair the intestinal mucosal barrier has become a fundamental approach to treating IBD. Studies have confirmed that the NF-κB signaling pathway and autophagy are mutually regulated, and that activation of the NF-κB signaling pathway can further activate autophagy and trigger inflammation (Cadwell, 2016; Piao et al., 2017). Therefore, inhibiting autophagy *via* NF-κB pathway inhibition is a potential therapeutic approach for treating IBD.

Huangkui lianchang decoction (HLD) is a traditional herbal formula consisting of six common herbs, *Huang Shu Kui Hua, DiJin Cao, Feng Wei Cao, Zi Cao, Qian Cao*, and *Wu Bei Zi*. In our previous clinical observation, we found that HLD enema reduced the erythrocyte sedimentation rate (ESR) and C-reactive protein (CRP) expression in patients with UC, showing an excellent anti-inflammatory effect. Further studies showed that HLD alleviates intestinal inflammation in UC mice by inhibiting the NF-κB signaling pathway (He et al., 2019); however, the effects of HLD on autophagy and the crosstalk between NF-κB and autophagy are unknown.

Here, we hypothesized that HLD exerts anti-UC effects by regulating autophagy *via* NF-κB pathway inhibition *in vitro* and *in vivo*. We evaluated the anti-inflammatory effect of HLD using a UC mouse model and verified its anti-UC effect exerted through autophagy and NF-κB pathway inhibition in intestinal epithelial cells. This study provides a scientific basis for preventing and treating UC using HLD.

## Materials and methods

### Main reagents and drugs

Mesalazine enemas (Salofalk, 60 g: 4 g, manufactured by Vifor AG Zweigniederlassung Medichemie Ettingen, Switzerland; import drug license number: H20150127) were purchased from Shenzhen Kangzhe Pharmaceutical Co. Dextran sodium sulfate (DSS), NF-κB p65, Beclin 1, p-NF-κB p65, LC3II, LC3I, and β-actin antibodies were purchased from Cell Signaling Technology (CST). Antibodies against 3-Methyladenine (3-MA), rapamycin (RAPA), and pyrrolidine dithiocarbamate (PDTC) were purchased from Malone Pharma Consulting.

### HLD preparation and quality control

*Abelmoschus manihot* (produced in Sichuan Province, China; lot No. 181015006), *Herba Euphorbiae humifusae* (produced in Jiangsu Province, China; lot No. 180801010), *Herba Pteridis multifidae* (produced in Jiangsu Province, China; lot No. 181004015), *Radix Arnebiae seu Lithospermi* (produced in Xinjiang Province, China; lot No. 180703), *Radix Rubiae* (produced in Shaanxi Province, China; lot No. 180617), and *Galla Chinensis* (produced in Guizhou Province, China; lot No. 180504005) were purchased from Suzhou Tianling Chinese Herbal Medicine Co., Ltd. All drugs were identified by the Suzhou Hospital of TCM pharmacist per Chinese Pharmacopoeia standards. Their corresponding English names, Latin names, plant names, and herb IDs were retrieved from http://herb.ac.cn and are listed in Table 1. Plant names were verified at http://www.theplantlist.org.

**TABLE 1:** Plant names and used parts of herbs included in the HLD.

Herb id	Chinese name	English name	Latin name	Plant name	Used parts
HERB 002574	*HUANG SHU KUI HUA*	*Setose Abelmoschus*	*Abelmoschus manihot*	*Abelmoschus manihot (L.) Medik*	flower
HERB 001254	*DI JIN CAO*	*all - grass of Humifuse Euphorbia*	*Herba Euphorbiae humifusae*	*Euphorbia humifusa Willd*	whole herb
HERB 001713	*FENG WEI CAO*	*all - grass of Chinese brake*	*Herba Pteridis multifidae*	*Pteris multifida Poir*	whole herb or rhizome
HERB 007160	*ZI CAO*	*Arnebia root Gromwell root*	*Radix Arnebiae seu Lithospermi*	*Arnebia euchroma (Royle) I.M. Johnst*	root
HERB 004446	*QIAN CAO*	*Tali Madder root*	*Radix rubiae*	*Rubia cordifolia L*	root
HERB 005692	*WU BEI ZI*	*Chinese gall*	*Galla Chinensis*	*Rhus chinensis Mill*	gall

The drugs were washed with water and soaked in distilled water equivalent to eight times the number of herbs for 60 min, boiled for 40 min, and extracted twice; the extracts were centrifuged at 3000 r/min for 30 min, concentrated to 2.5 g/ml liquid, sterilized with flow-through steam at 100°C for 30 min, and stored at 4°C.

### UHPLC- MS analysis

HLD was separated on an EXIONLC System (Sciex Technologies, United States) ultra-performance liquid chromatography using a Waters Acquity HSS T3 column (1.8 μm 2.1 × 100 mm) at a flow rate of 0.4 ml/min. Mobile phases A and B were aqueous solutions containing 0.1% formic acid and acetonitrile, respectively. Gradient elution was performed as follows: 0–0.5 min, 2% B; 0.5–10 min, 2–50% B; 10–11 min, 50–95% B; 11–13 min, 95% B; 13–13.1 min, 95–2% B; 13.1–15 min, 2% B. The column temperature was 40°C. The autosampler temperature was 4°C, and the injection volume was 2 μl.

Sciex Q Trap 6500+ (Sciex Technologies) was used for the assay development. Typical ion source parameters were: ion spray voltage: +5500 V, curtain gas: 35 psi, temperature: 400°C, ion source gas 1:60 psi, ion source gas 2: 60 psi, DP: ± 100 V. The SCIEX Analyst Work Station Software (Version 1.6.3) was employed for multiple reaction monitoring data acquisition and processing.

### Model preparation and drug delivery

**FIGURE 1:**
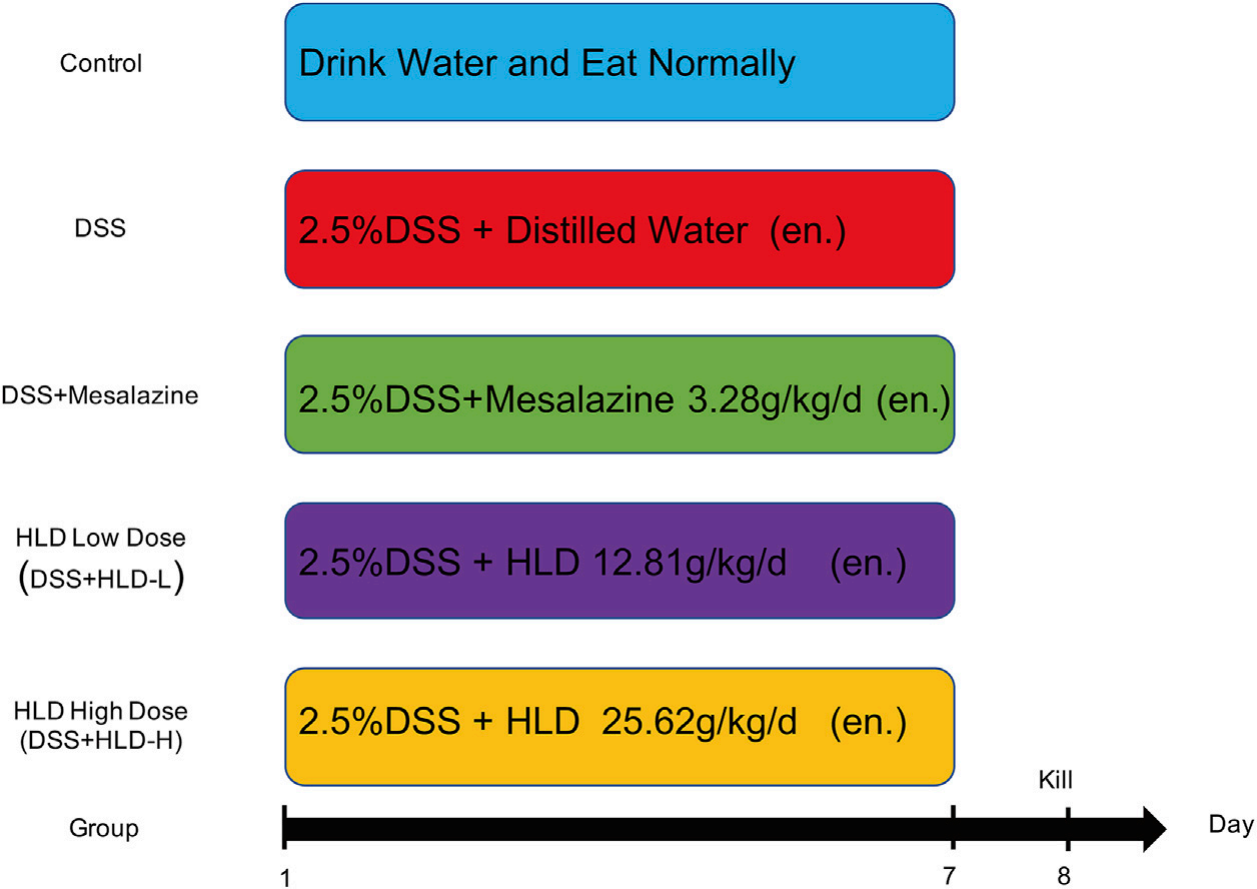
Experimental flow and drug delivery scheme.

Fifty 8-week-old C57BL/6 male mice were purchased from Charles River Laboratories (Beijing, China). Mice were acclimatized and housed for 1 week on a 12 h light/12 h dark cycle. The mouse UC model was established referring to previous literature (Murano et al., 2000; Wang et al., 2018; Peng et al., 2020). In brief, DSS was dissolved in sterile water to form a 2.5% DSS (36,000–50,000 Da) solution, and mice drank continuously for 7 days. The control group was administered saline. Mice were divided into five groups (10 mice in each group) by the random number table method as follows: Control group, DSS group, Mesalazine group (DSS+Mesalazine), HLD low-dose group (DSS+HLD-L), and HLD high-dose group (DSS+HLD-H). The modeling protocol and procedure are shown in Figure 1. The study was approved by the Medical Ethics Committee of Suzhou Hospital of TCM (Grant No. 2020 Ethical Animal Approval 002) before implementation. The research was conducted per the Directive 2020/63/EU to protect the laboratory animals. The study followed the ARRIVE guidelines 2.0 for the design, analysis, and reporting. The experimental operators were aware of the group distribution, experiment conduct, and outcome assessment during group allocations. Data analysts were not aware of group allocations.

### Anesthesia and enema method

According to the manufacturer’s instructions, mice were anesthetized with isoflurane, an inhalational anesthetic, using an ABS-type small animal anesthesia machine. Anesthetized mice were placed in the prone position. The drug was injected slowly into the intestinal lumen with a 1 ml syringe and a 20G cannula needle (lubricated with Vaseline), the top of which was approximately 4 cm from the anus. The abdomen was gently rubbed for 1 min and then placed restraining the mouse head down, and the body was tilted by 45° for 10 min, once a day.

### Disease activity index (DAI) score

The disease activity index (DAI) was determined as described previously (Murano et al., 2000; Wang et al., 2018; Peng et al., 2020). Body weight loss, stool haemoccult positivity or gross bleeding, and stool consistency were evaluated according to Table 2. DAI was calculated with the following formula: DAI = (combined score of weight loss, stool consistency, and bleeding)/3.

**TABLE 2:** DAI scores in mice.

Score	Weight loss (%)	Stool consistency	Occult/gross bleeding
0	None	Normal	None
1	1–5	Soft, shaped	Between the two
2	6–10	dilute stool	Fecal Occult Blood
3	11–15	Between the two	Between the two
4	>15	Diarrhea	Blood in the stool

DAI = (combined score of weight loss, stool consistency, and bleeding)/3, 5% body weight loss was scored as 1. On the modeling day, the body weight was used as the basal body weight.

### Histological scoring

On day eight of the experiment, the mice were euthanized, and blood and colonic tissue specimens were collected according to standard operating procedures. Distal colon tissues with significant inflammation/ulcer were fixed in a 4% paraformaldehyde solution, embedded in paraffin, serially sectioned at 4 μm, and stained with hematoxylin-eosin (HE). The inflammatory infiltration and colonic mucosal damage degree were observed using CaseViewer, a digital microscopy application, and histological scores were obtained. Histological scoring was performed according to the following criteria: epithelium: 0, normal form; 1, loss of cup cells; 2, significant loss of cup cells; 3, loss of crypt; 4, significant loss of crypt and inflammatory infiltration: 0, no infiltration; 1, infiltration at the base of the crypt; 2, infiltration into the mucosal muscular layer; 3, extensive infiltration into the mucosal muscular layer with mucosal thickening and edema; 4, infiltration into the submucosal layer. The total histological score included the sum of the epithelial and inflammatory infiltration scores.

### ELISA

According to the standard experimental procedure, ELISA kits were used to detect NF-κB and TGF-β concentrations in mouse serum and TNF-α, IL-1β, IL-6, and IL-10 concentrations in mouse serum and cell supernatants.

### Immunohistochemistry

The preserved paraffin sections were analyzed *via* microscopy following the dewaxing, rehydration, fire extinguishing, closure, secondary antibody labeling, incubation, diaminobenzidine (DAB) color development, and hematoxylin re-staining and sealing process. Five high-magnification fields (1000×) were selected for each section and analyzed after quantitative grayscale scanning using the image analysis system. NF-κB p65 expression and localization in the cytoplasm and nucleus and autophagy-related protein (LC3 and Beclin 1) expression in colon tissue sections were detected using immunohistochemistry.

### Immunofluorescence

After deparaffinization and rehydration, distal colon sections were microwaved in citric acid (pH 6.0). After washing with phosphate-buffered saline containing Tween-20 (pH 7.4), the sections were incubated in a 3% H_2_O_2_ solution and blocked with 3% bovine serum albumin for 20 min at room temperature (Zhao et al., 2020). The sections were then incubated with NF-κB p65 (1:100; CST, Danvers, MA, United States), LC3 (1:200; CST, Danvers, MA, United States), and Beclin 1 (1:150; CST, Danvers, MA, United States) primary antibodies at 4°C overnight. The blots were incubated with horseradish peroxidase (HRP)-conjugated secondary antibody (1:200; Servicebio, China) for 30 min at room temperature, followed by DAB solution (Servicebio, China). The sections were counterstained with hematoxylin, dehydrated, and mounted. Sections were observed using Caseviewer and Leica LAS image acquisition systems. Positive DAB staining areas were quantified using the NIH ImageJ software (National Institutes of Health, Bethesda, MD, United States).

### HLD-DS preparation

Sprague Dawley rats were administered HLD 1.5 ml/time (five times the adult equivalent dose) twice a day for three consecutive days. One hour after the last administration, the rats were sacrificed by carbon dioxide asphyxiation, and blood was drawn from the abdominal aorta. The serum was aseptically separated by centrifugation at 2500 rpm for 10 min, inactivated at 56 C for 30 min, and filtered through a 0.22 μm microporous membrane to remove bacteria. Blood was stored at −80°C.

### Cellular experiments

NCM460 human colonic epithelial cells were purchased from Shanghai Meiwan Biotechnology Co., Ltd and grown in RPMI medium containing penicillin (100 U/ml)-streptomycin (100 U/ml) (Invitrogen, Carlsbad, CA, United States) and 10% fetal bovine serum (Hyclone, Logan, UT, United States) (Zhou et al., 2018). Lipopolysaccharide (LPS, 10 ng/ml) was used to induce inflammation and autophagy in NCM460 cells (Zeng et al., 2018; Ba et al., 2021; Guo et al., 2021; Shang et al., 2021). The groups were divided into control, LPS, LPS+HLD-DS, LPS+HLD-DS+3-MA, LPS+HLD-DS+RAPA, LPS+HLD-DS+PDTC, LPS+HLD-DS+PDTC+RAPA, and LPS+HLD-DS+PDTC+3-MA. The concentrations of 3-MA, RAPA, and PDTC were 100 nmol/L, 3 mmol/L, and 50 µmol/L, respectively.

### Cell counting kit-8 (CCK-8) assay

According to the manufacturer’s protocol, the effect of different HLD-DS concentrations on cell proliferation was examined using CCK-8. NCM460 cell suspension with a density of 1 × 10^4^ cells/ml was inoculated into a 96-well plate (100 µl per well) and incubated at 37°C for 4 h. Cells were treated with 10 µl of CCK-8 and incubated for 4 h. The OD value at 450 nm was measured, and the measurement of each sample was repeated six times.

### Western blotting

Total cellular proteins were extracted using a Radio Immunoprecipitation Assay buffer, and protein concentrations were determined using the bicinchoninic acid (BCA) method. Equal amounts of proteins were electrophoresed on 10% sodium dodecyl sulfate-polyacrylamide gels by electrophoresis, followed by immunoblotting to transfer the proteins to polyvinylidene fluoride (PVDF) membranes. PVDF membranes were blocked with skim milk for 2 h at room temperature and incubated with LC3II and LC3I (1:1000; #12741s, CST), Beclin1 (1:1000; #4122, CST), NF-κB p65 (1:1000; #6956s, CST), p-NF-κB p65 (1:1000; #3033, CST), and β-actin (1:1000; #3700, CST) antibodies overnight at 4°C. After incubation with HRP-conjugated secondary antibodies, proteins were detected by electrochemiluminescence (ECL) using the ChemiDoc CRS imaging system and analyzed using Quantity One analysis software (Bio-Rad Laboratories, San Francisco, CA, United States).

### Data analysis and graphing

All data are presented as mean ± standard deviation (SD) from a minimum of three replicates. Differences between the groups were evaluated using GraphPad Prism version 9.0.0 (GraphPad Software, San Diego, California, United States) with Student’s *t*-test when comparing only two groups or assessed by one-way ANOVA when more than two groups were compared. Differences were considered statistically significant at *p* < 0.05.

## Results

### Chemical characteristics of HLD

**FIGURE 2:**
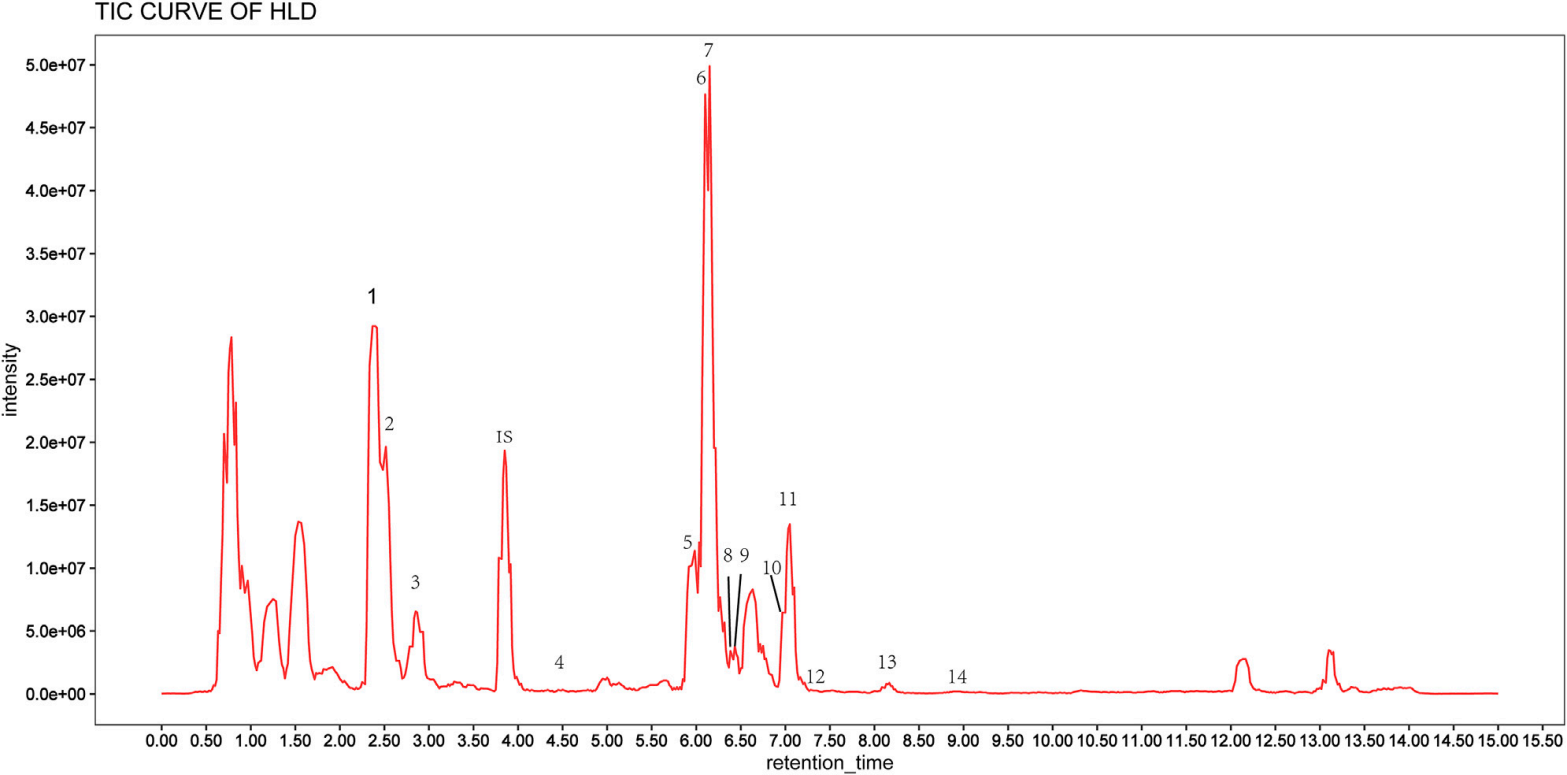
LC-MS chromatogram of HLD.

An ultra-high performance liquid chromatography-tandem mass spectrometry (UHPLC-MS/MS) method was developed to characterize 14 compounds in HLD. The base peak chromatograms of HLD are shown in Figure 2. The compounds were identified by literature comparison and mass spectrometry, and the results are presented in Table 3. The results indicated that HLD mainly contained flavonoids, such as rutin, isoquercitrin, gossypetin, and quercetin. These flavonoids were present in more than one herb.

**TABLE 3:** HLD bioactive components.

Number	Components	Rt (min)	Formula	MS	MS^2^
1	2-Phenylacetamide	2.52	C_8_ H_9_ NO	135.0684	119
2	Inosine	2.60	C_10_ H_12_ N_4_ O_5_	268.0807	137
3	L-Phenylalanine	2.82	C_9_ H_11_ NO_2_	165.0789	120.1
4	Cianidanol	4.50	C_15_ H_14_ O_6_	290.0790	165
5	Rutin	5.85	C_27_ H_30_ O_16_	610.1534	465
6	Cynaroside	6.10	C_21_ H_2_0 O_11_	448.1005	286.9
7	Isoquercitrin	6.22	C_21_ H_2_0 O_12_	464.0954	303
8	Gossypetin	6.46	C_15_ H_10_ O_8_	318.0375	109
9	Rhoifolin	6.50	C_27_ H_30_ O_14_	578.1636	433
10	Hyperoside	6.99	C_21_ H_2_0 O_12_	464.0955	302.8
11	Myricetin	7.05	C_15_ H_10_ O_8_	318.0376	272.9
12	Alizarin 2-methyl ether	7.38	C_15_ H_10_ O_4_	254.2245	237.15
13	Quercetin	8.07	C_15_ H_10_ O_7_	302.0426	153
14	Lucidin	8.89	C_15_ H_10_ O_5_	270.0528	253.1

### HLD improved the DAI and alleviated the pathology of UC mice

**FIGURE 3:**
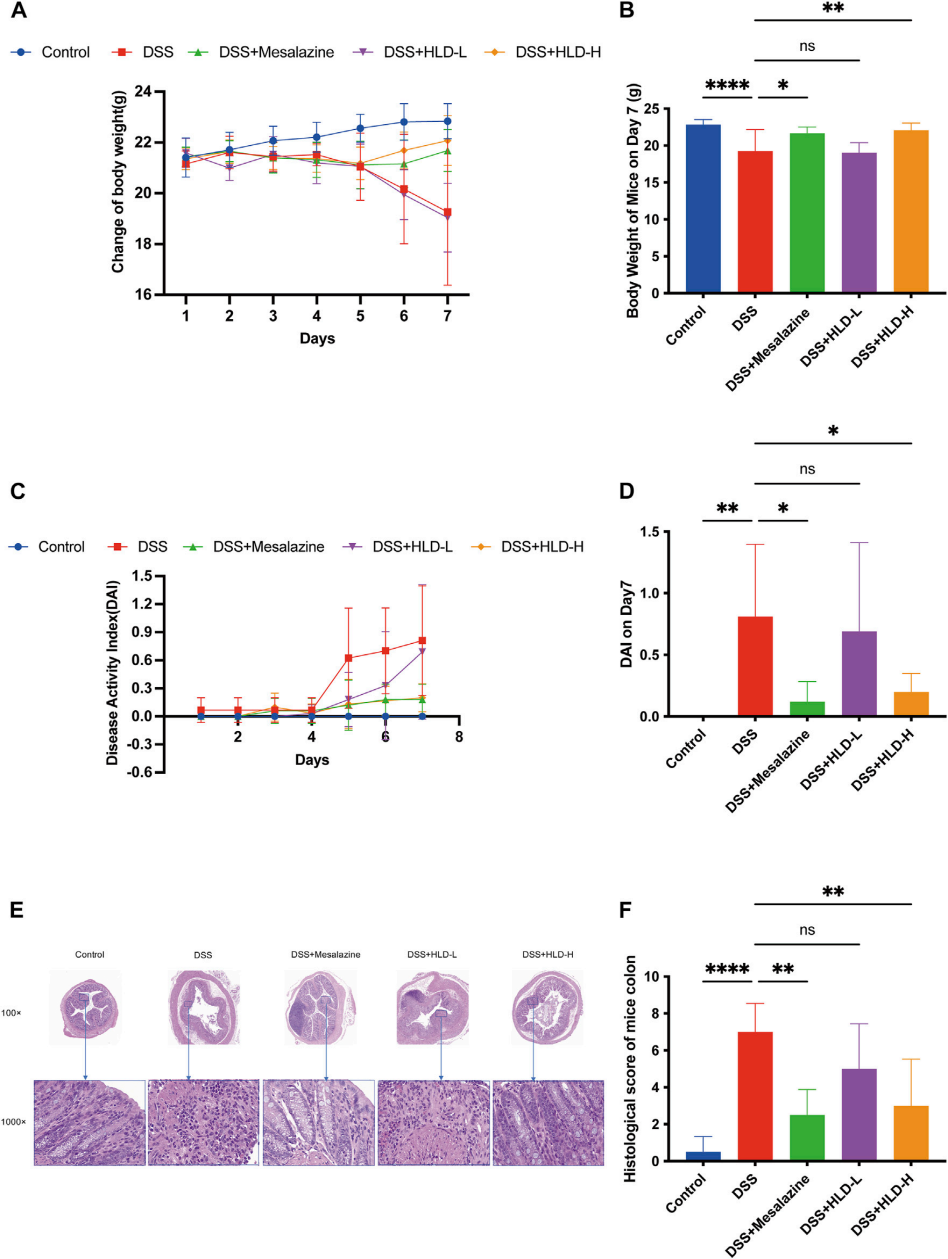
HLD improved DAI and alleviated the UC mice’s pathology. **(A**,**B)** The trend of body weight changes in mice and a comparison of body weight on day 7 were presented. **(C**,**D)** The trend of DAI changes in mice and a comparison of DAI on day 7 were presented. **(E**,**F)** The comparison of hematoxylin & eosin (HE) staining results and histological scores of mice colonic tissues. All data are expressed as the mean ± SD. (*n* = 3). *p < 0.05, **p < 0.01, ****p < 0.0001.

DSS-induced UC mice exhibited persistent weight loss from day 4, accompanied by diarrhea and fecal occult blood (Figure 3A). By day 7, the weights of the DSS group mice were significantly lower than those of the control mice (*p* < 0.0001) (Figure 3B) and were accompanied by fecal blood. The weights of the mesalazine and DSS+HLD-H groups decreased slightly and then gradually increased. On the seventh day, the body weights of the mesalazine and DSS+HLD-H groups were significantly different from those of the DSS group (Figure 3B). One mouse in each of the DSS and HLD-H groups died during the experiment, and five mice died in the Mesalazine group. No death was observed in the remaining groups.

The DAI scores of the DSS group mice were significantly higher than those of the control group (*p* < 0.01) (Figure 3D). The DAI scores of the mesalazine and DSS+HLD-H groups were significantly lower than those of the DSS group (*p* < 0.05). In contrast, the DAI score of the DSS+HLD-L group was not significantly different from that of the DSS group (Figure 3D).

As shown in Figure 3E, the control group showed a clear colonic tissue structure in all layers, intact mucosal epithelium, tightly arranged crypts, regular muscle fibers arrangement in the muscular layer, and no apparent inflammatory reaction. The DSS group showed extensive ulcer formation. Many inflammatory cells infiltrated the mucosal and submucosal layers. Dilated intestinal glands and a few acidophilic or basophilic masses in the glandular lumen were observed, suggesting successful modeling. The inflammatory reaction, ulceration, submucosal edema, and plasma membrane layer inflammation were less severe in the mesalazine and HLD-H groups compared with the DSS group. The HLD-L group showed intestinal mucosa ulceration, numerous inflammatory cells in the mucosal and submucosal layers, and bleeding in the intestinal lumen in some samples. There was a significant difference in histological scores between the mesalazine and HLD-H groups compared with the DSS but not the HLD-L group (Figure 3F).

### HLD exerts anti-inflammatory effects by inhibiting NF-κB *in vivo*

**FIGURE 4:**
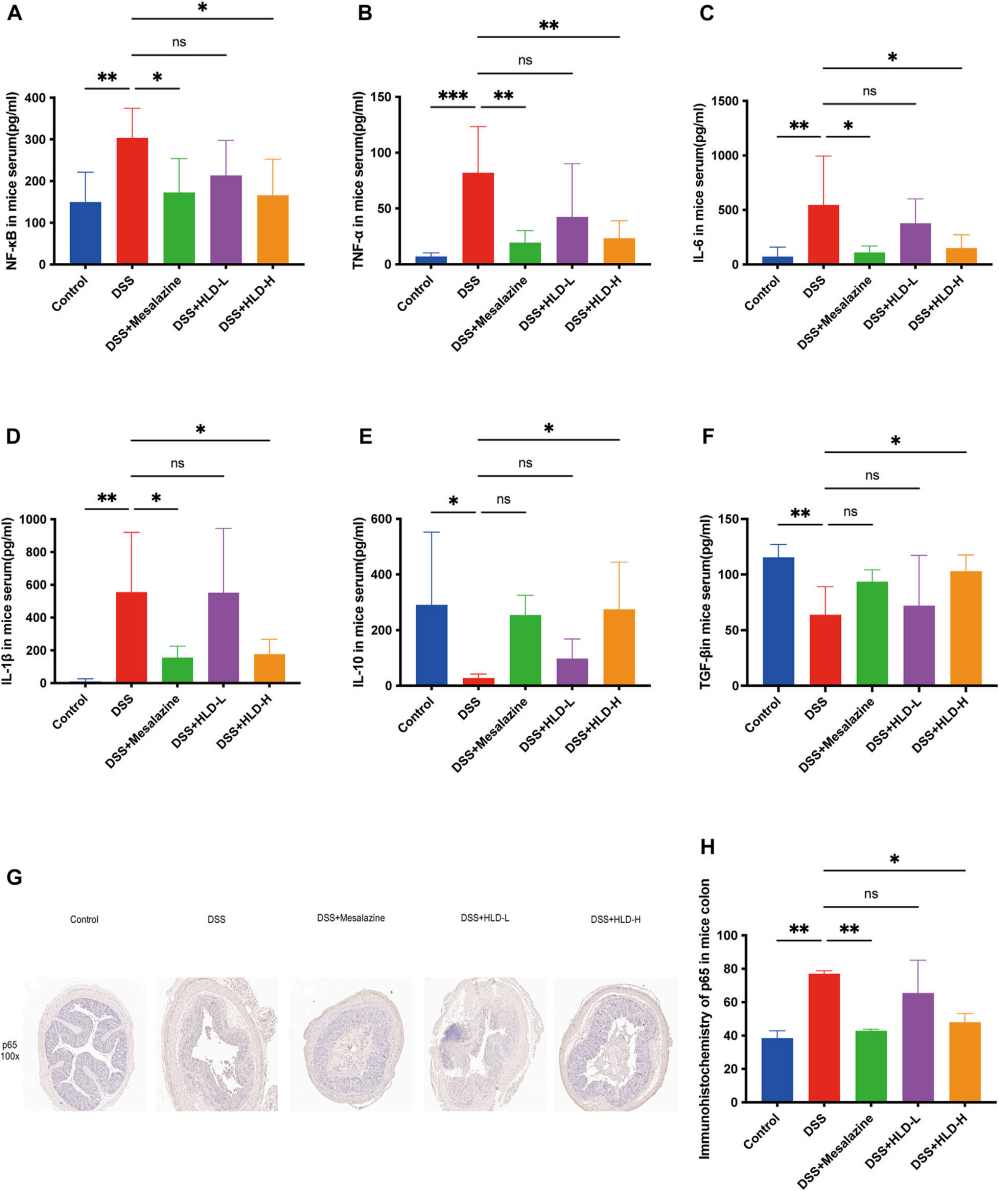
HLD exerted anti-inflammatory effects by inhibiting NF-κB *in vivo*. **(A**–**F)** The levels of pro-inflammatory cytokines such as NF-κB, TNF-α, IL-6, IL-1β and anti-inflammatory factors such as TGF-β and IL-10 were detected by ELISA in response to the anti-inflammatory activities of HLD. (*n* = 6). (**G**,**H**) Immunohistochemical sections and data comparison of NF-κB p65 in mice colonic tissues. All data were expressed as the mean ± SD. (*n* = 3). *p < 0.05, **p < 0.01, ***p < 0.001.

As shown in Figure 4 A-F, TGF-β and IL-10 levels were significantly reduced in the DSS group compared to the control group, and NF-κB, TNF-α, IL-6, and IL-1β levels were significantly increased in the DSS group. The HLD-H group showed significantly increased TGF-β and IL-10 levels and reduced NF-κB, TNF-α, IL-6, and IL-1β levels compared to the DSS group (*p* < 0.05).

Furthermore, NF-κB p65 was deeply stained in the nucleus during the apparent colonic tissue inflammation in the DSS group (Figure 4G). NF-κB p65 expression decreased in the colon tissues of the HLD-H and mesalazine groups, suggesting that HLD exerted an anti-inflammatory effect by inhibiting NF-κB (Figure 4H).

### HLD exerts anti-inflammatory effects by inhibiting autophagy *in vivo*

**FIGURE 5:**
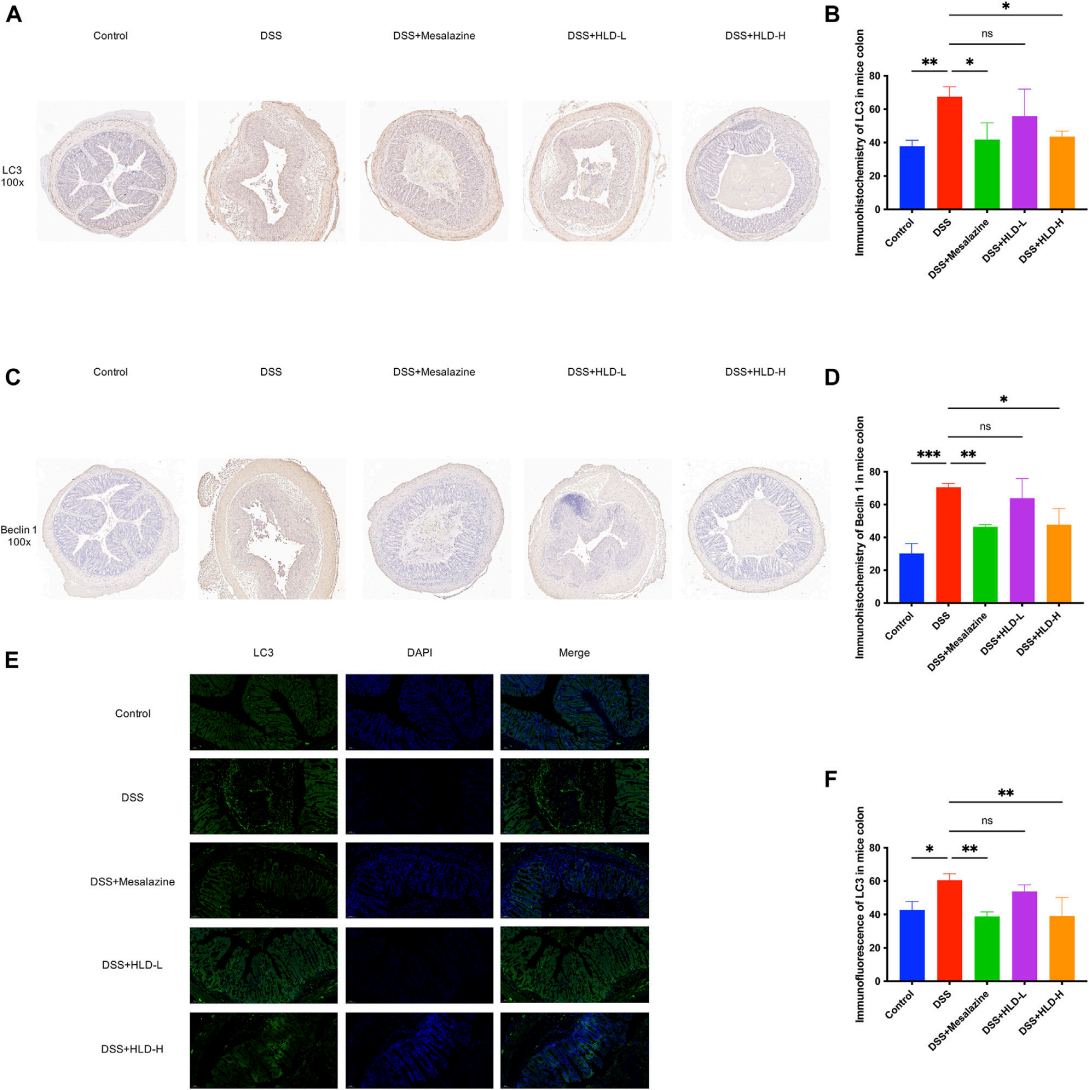
HLD exerted anti-inflammatory effects by inhibiting autophagy *in vivo*. **(A**,**B)** Comparison of immunohistochemistry of LC3 in mice colonic tissues. **(C**,**D)** Comparison of immunohistochemistry of Beclin 1 in mice colon tissues. **(E**,**F)** LC3 expression in mice colonic tissues detected by immunofluorescence. All data were expressed as the mean ± SD. (*n* = 3). *p < 0.05, **p < 0.01, ***p < 0.001.

On comparing LC3 immunohistochemical sections and immunofluorescence staining pictures of colonic mouse tissues, LC3 expression was significantly higher in the DSS group than that in the control group, with a statistically significant difference. This suggested that autophagic activity was significantly higher when colonic inflammation was active (Figures 5A,B,E,F). Compared with the DSS group, LC3 was significantly lower in the mesalazine and HLD-H groups, suggesting a decrease in autophagic activity (Figures 5A,B,E,F). A comparison of Beclin 1 immunohistochemical sections yielded results consistent with LC3 (Figures 5C,D).

### HLD-DS CCK-8 experiment

**FIGURE 6:**
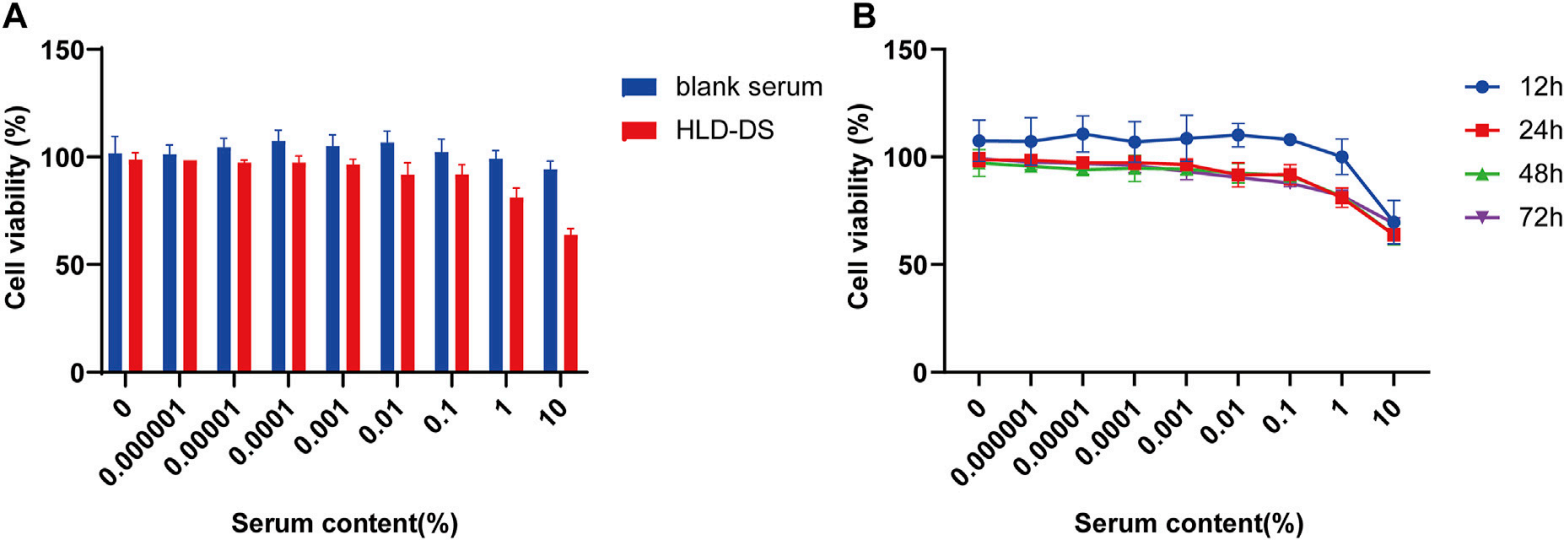
HLD-DS CCK-8 experiment. The cytotoxicity of NCM460 cells treated by HLD-DS was investigated by the CCK-8 assay. **(A)** Effects of serial concentrations of HLD-DS on cell viability. **(B)** The effect of 1% HLD-DS on cell viability at 12, 24, 48, and 72 h. All data are presented as mean ± SD. (*n* = 6).

NCM 460 cells are immortalized cells isolated from normal human tissues and are a common cell model for studying various biological colon functions. As shown in Figure 6, there was no significant difference in cell survival at a concentration of 1% HLD-DS upon exposure for 24 h when compared with the same concentration of blank serum. There was a significant difference in cell viability with up to 10% HLD-DS concentration. Therefore, 1% HLD-DS was chosen as the optimal concentration.

### HLD-DS alleviates LPS-induced inflammation in NCM460 cells by inhibiting the NF-κB pathway and autophagy

**FIGURE 7:**
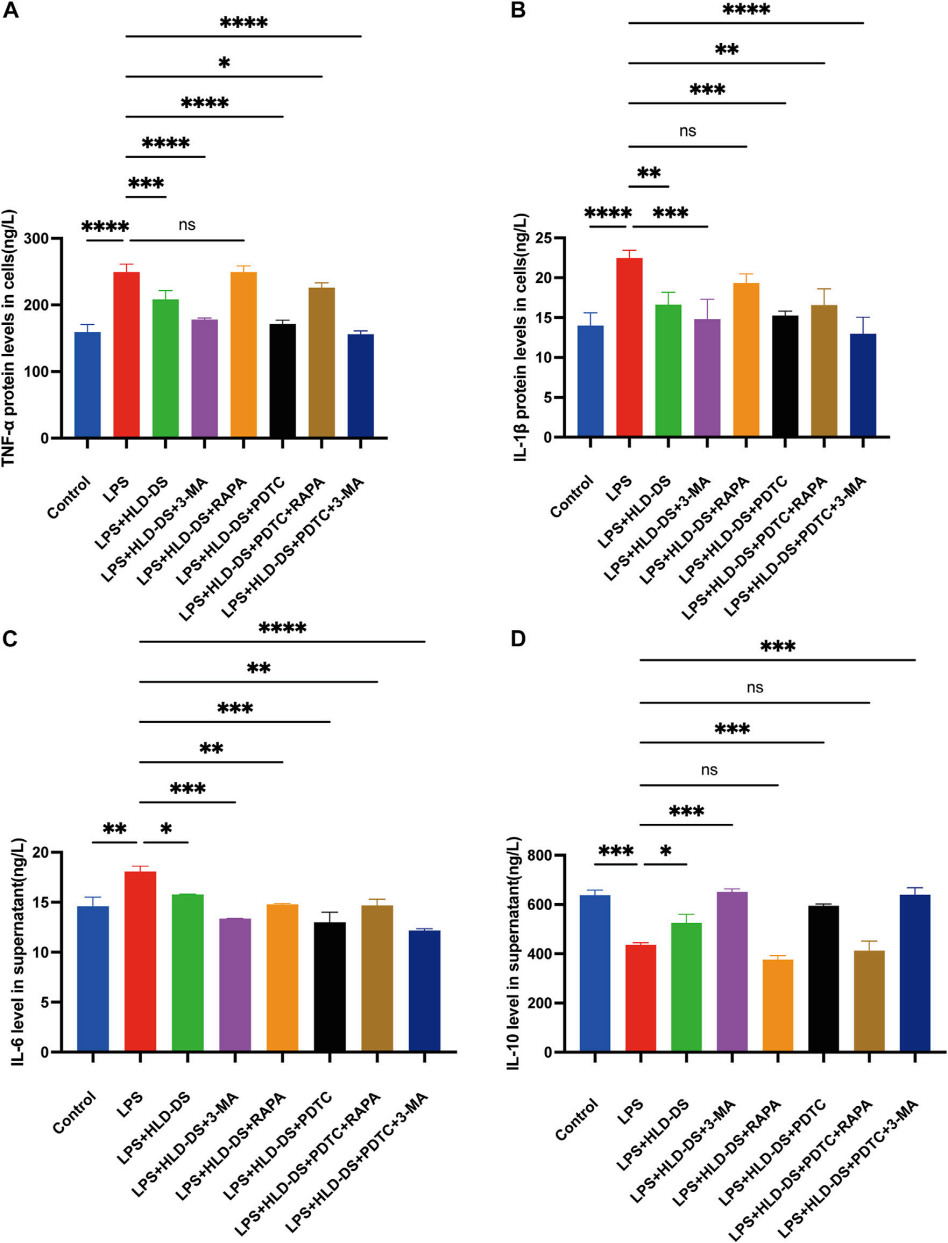
HLD alleviated LPS-induced inflammation in NCM460 cells by inhibiting the NF-κB pathway and autophagy. (**A**–**D**) TNF-α, IL-6, IL-1β, and IL-10 levels in the cell supernatant were detected by Elisa. All data are expressed as the mean ± SD. (*n* = 3). *p < 0.05, **p < 0.01, ***p < 0.001, ****p < 0.0001.

ELISA results showed that the inflammatory response was evident after being induced by LPS in the NCM460 cells (Figure 7). Compared to that in the control group, TNF-α, IL-1β, and IL-6 expressions were elevated in the cell culture medium, and IL-10 expression was significantly reduced when induced by LPS. The difference was statistically significant, suggesting that the cells were modeled successfully. Compared with the LPS group, the levels of TNF-α, IL-1β and IL-6 were significantly decreased in the LPS + HLD-DS group, and the level of IL-10 was significantly increased. This indicated that HLD-DS could inhibit inflammation in UC significantly. It was also observed that TNF-α, IL-1β, and IL-6 were significantly decreased, and IL-10 was significantly increased when LPS-induced NCM460 cells were exposed to HLD-DS with 3-MA or PDTC simultaneously. However, RAPA reduced the effect of HLC-DS on reducing TNF-α, IL-1β, and IL-6 and increasing IL-10. These results suggested that HLD-DS protected against LPS-induced inflammation in NCM460 cells by inhibiting the NF-κB pathway and autophagy.

### HLD-DS inhibited autophagy induced by LPS in NCM460 cells

**FIGURE 8:**
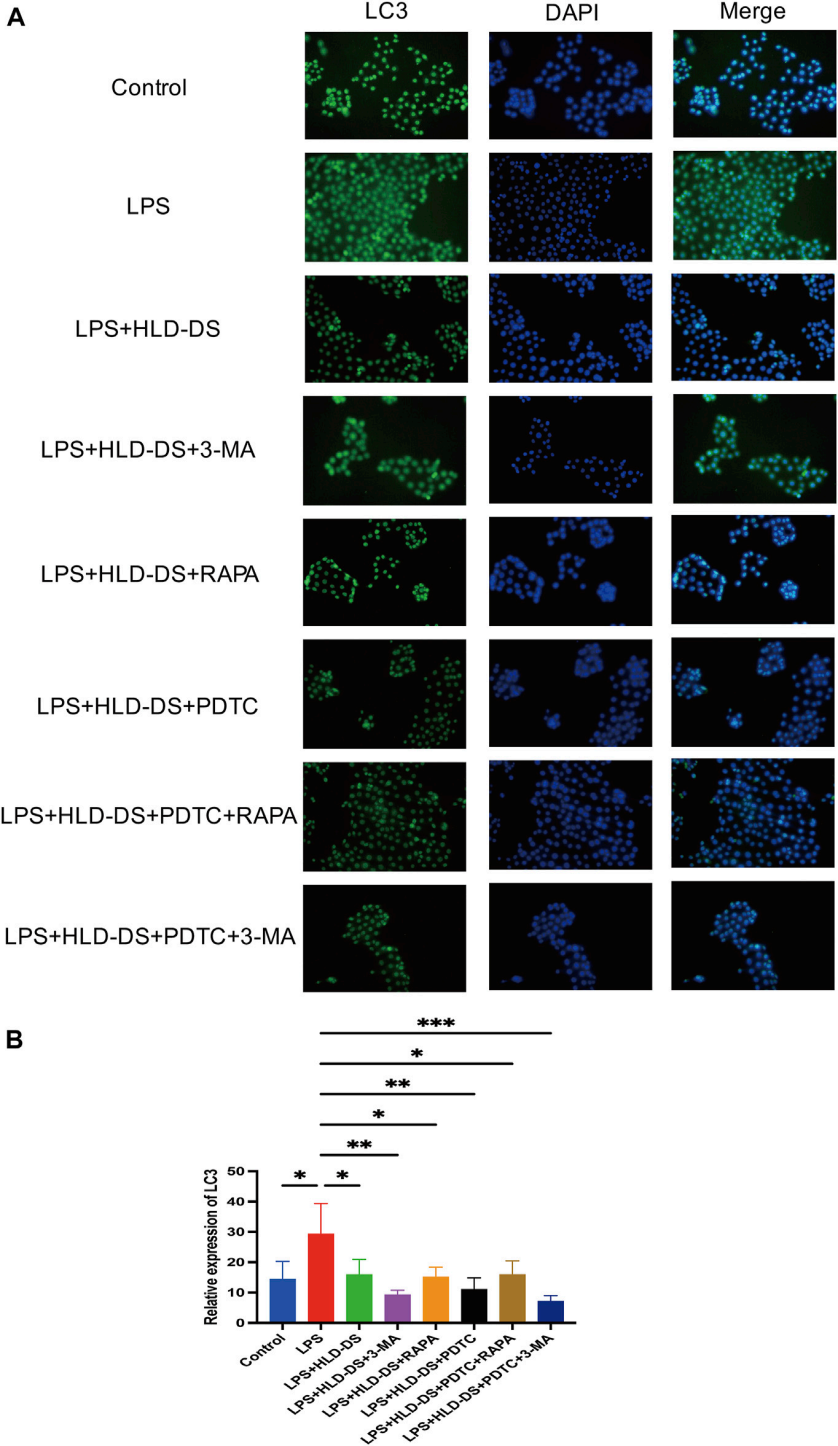
HLD-DS inhibited expression of LC3 in cells detected by immunofluorescence. **(A)** LC3 expression was detected by immunofluorescence in LPS-induced NCM-460 cells. **(B)** Quantitative results of LC3 protein expression in LPS-induced NCM460 cells. All data are expressed as the mean ± SD. (*n* = 3). *p < 0.05, **p < 0.01, ***p < 0.001.

Immunofluorescence results showed that LC3 expression was significantly enhanced after induction by LPS in NCM460 cells (Figure 8). Compared with the LPS group, the LC3 level in the LPS+HLD-DS group was significantly decreased, indicating that HLD-DS could significantly inhibit autophagy. When LPS-induced NCM460 cells received HLD-DS and 3-MA or PDTC, LC3 expression was significantly reduced. Conversely, RAPA can increase LC3 expression. These results suggested that HLD-DS could inhibit LPS-induced autophagy in NCM460 cells.

### HLD inhibited the NF-κB pathway and autophagy in LPS-induced NCM460 cells

**FIGURE 9:**
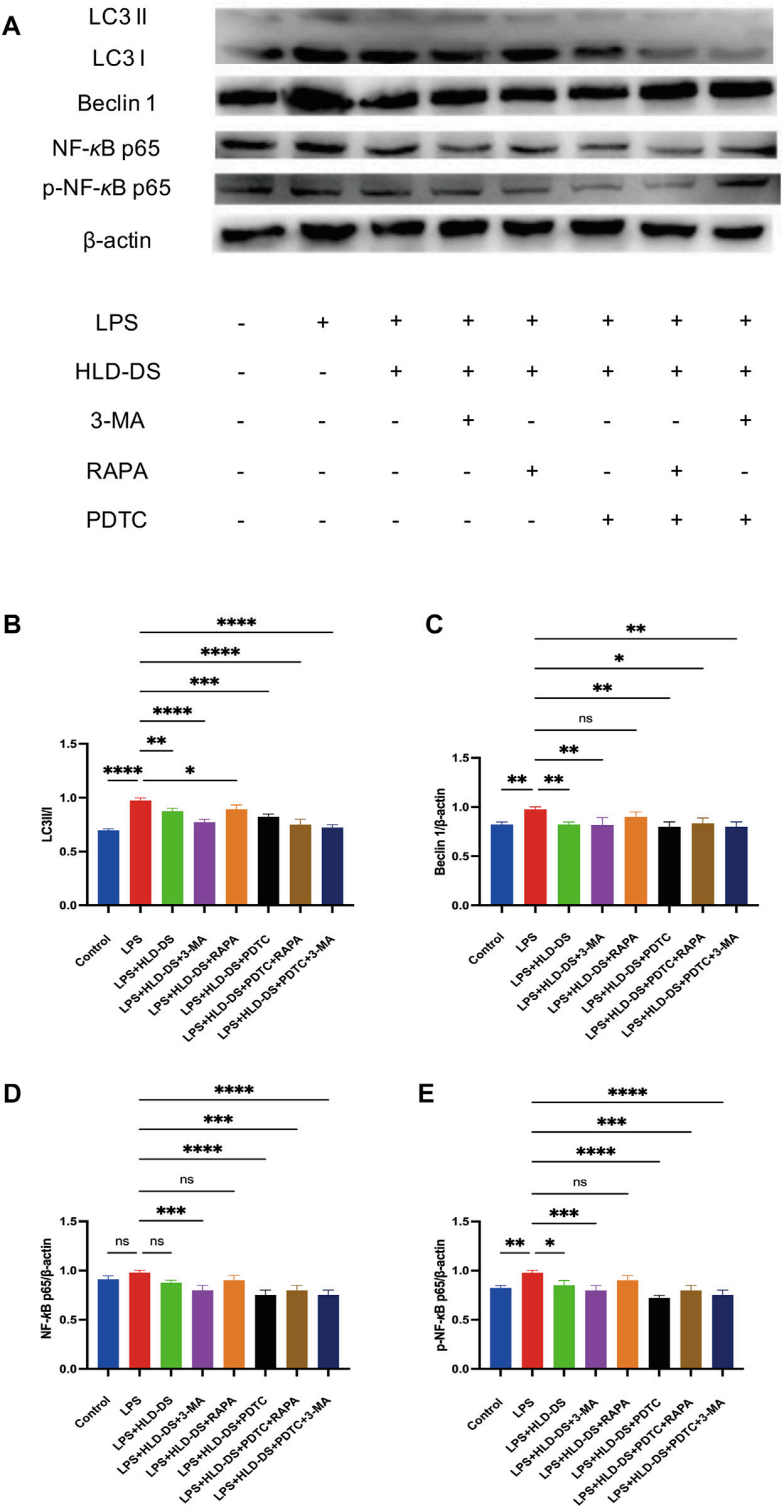
HLD-DS affected protein expressions related to the NF-κB pathway and autophagy. **(A)** Western blots of LC3I, LC3II, Beclin 1, NF-κB p65, and p-NF-κB p65 in LPS-induced NCM-460 cells. **(B)** Quantitative results of LC3II/LC3I protein expression in LPS-induced NCM460 cells. **(C)** Quantitative results of Beclin I protein expression in LPS-induced NCM460 cells. **(D**,**E)** Quantitative results of NF-κB p65 and p-NF-κB p65 protein expression in LPS-induced NCM460 cells. All data are expressed as the mean ± SD. (*n* = 3). *p < 0.05, **p < 0.01, ***p < 0.001, ****p < 0.0001.

Western blotting showed that p-NF-κB p65 expression was significantly higher in the LPS group than that in the control group (Figure 9). Compared to LPS expression, p-NF-κB p65 expression was significantly reduced in the LPS+HLD-DS and LPS+HLD-DS+PDTC groups. The LPS+HLD-DS+PDTC group was superior to the LPS+HLD-DS group (*p* < 0.05). These results suggest that HLD-DS protects against LPS-induced NCM460 cells by inhibiting the NF-κB pathway. LC3II/I and Beclin 1 protein expressions were significantly higher in the LPS group than in the control group. Compared to that in the LPS group, LC3II/I and Beclin 1 protein expression in the LPS+HLD-DS group decreased. The changes in LC3II/I and Beclin 1 protein expression in the LPS+HLD-DS+PDTC group were not apparent after the addition of the NF-κB pathway inhibitor, PDTC, compared to the LPS+HLD-DS group.

These results suggested that HLD-DS inhibited LPS-induced inflammation in NCM460 cells. The underlying mechanisms were found as the NF-κB pathway and autophagy. However, there was no obvious crosstalk between the NF-κB pathway and autophagy.

## Discussion

The NF-κB pathway is essential for IBD development and progression. HLD has been used clinically for several years to treat mild-to-moderate UC with good efficacy. HLD is a traditional Chinese medicine preparation with clinical efficacy for UC treatment. This study verified for the first time that HLD could simultaneously inhibit autophagy and the NF-κB pathway to alleviate DSS-induced colitis in mice, although with no apparent crosstalk between autophagy and the NF-κB pathway.

The synergistic effects of multiple components are a characteristic of herbal medicines (Rao et al., 2022; Yu et al., 2022). HLD complexity was demonstrated by characterizing 14 major compounds, including flavonoids, phenols, and alkaloids, using UHPLC-MS/MS. *Abelmoschus manihot* is the main HLD drug. The results showed that it contains a variety of flavonoids such as isoquercitrin, hyperin, myricetin, and quercetin. Recent data have suggested that flavonoids have significant efficacy in UC treatment. Pepper extract consisting of rutin, quercetin, and isoquercitrin attenuated colonic length shortening and pathological injury in a DSS-induced UC model. In addition, ZBE inhibited TNF-α, IL-1β, and IL-12 by regulating the TLR4-and TLR4-related pathways in mice and LPS-induced cellular inflammation in DSS-induced experimental colitis (Zhang et al., 2016). Flos lonicerae flavonoids (mainly hyperin, loniceraflavone, and luteolin) attenuated TBNS-induced UC by inhibiting the NF-κB pathway (Liu et al., 2020). Myricetin improved the severity of acute UC, increased IL-10 and TGF-β levels, and increased regulatory T cell proportion (Qu et al., 2020). *Alhagi pseudalhagi* extract containing hyperin, rutin, kaempferol, and isorhamnetin may protect against intestinal inflammation in UC by affecting the TLR 4-dependent NF-κB signaling pathway (Xu et al., 2021). Therefore, the synergistic effect of multiple components may be responsible for the therapeutic effect of HLD in UC.

This study found that high-dose HLD protected mice colonic tissues with DSS-induced colitis, as evidenced by reduced DAI and colonic histological scores, reduced NF-κB pathway-related inflammatory cytokines in mouse serum, and reduced NF-κB p65 levels in colonic tissues. More importantly, increased p-NF-κB p65 protein expression was observed in the LPS-induced cell models, and HLD-DS treatment reduced p-NF-κB p65 expression. These results further confirmed that HLD protects against DSS-induced colitis in mice and LPS-induced inflammation in NCM460 cells by inhibiting the NF-κB pathway. NF-κB pathway-related pro-inflammatory cytokines such as TNF-α, IL-1β, and IL-6 have long been identified as targets for anti-inflammatory strategies in UC. The expression of these pro-inflammatory cytokines was significantly elevated in the DSS-induced mice colitis and was significantly decreased after Anneslea fragrans Wall. and SYD interventions, followed by remission of colitis (Deng et al., 2021; Wei et al., 2021). These were consistent with the results of this study. The present study revealed that HLD protects against LPS-induced inflammation in NCM460 cells by inhibiting autophagy. We observed that autophagy-related LC3 and Beclin 1 protein expression was significantly increased *in vitro*; HLD-DS reversed this phenomenon, and the effect was more evident after adding the autophagy inhibitor 3-MA. Proteins LC3 and Beclin 1 were the most commonly used assays in autophagy-related studies (Haq et al., 2019). Herbal prescription Jianpi Qingchang Decoction significantly reduced LC3 and Beclin 1 protein expression and improved colitis in DSS-induced UC mice (Dai et al., 2017).

The regulation of autophagy and the NF-κB pathway has been demonstrated in malignant tumors, cardiovascular diseases, and bone and joint diseases (Feng et al., 2016; He et al., 2017; Qi et al., 2020; Wang and Gao, 2021). The related roles of autophagy and NF-κB pathway in HLD anti-UC were explored in this study. However, there revealed no clear regulatory relationship between the NF-κB pathway and autophagy. NF-κB transactivates many autophagy-related genes (eg, Beclin 1); thus, NF-κB-related inflammatory response signaling pathways overlap with those of autophagy. Normally, NF-κB in the cytoplasm binds to its inhibitor IκB, and when activated by the IκB kinase (IKK) complex, the displaced IκB is degraded by the proteasome. This activated NF-κB translocated into the nucleus to activate the inflammatory response. However, TGF-β-activating kinase 1 (TAK1) and its cofactors TAB2 and TAB3 were required for IKK activation. Because TAB2/TAB3 could also form a complex with Beclin 1, NF-κB activation could only result from changes in these balances and occurs concurrently with the autophagic response (Simon et al., 2017). This study confirmed that HLD exerted anti-UC effects by inhibiting autophagy and NF-κB pathways with non-crosstalk in UC. It was also shown that NF-κB couldn’t be involved in the induction of autophagy in the canonical NF-κB pathway. It was IKKα activity rather than NF-κB that controlled the basal expression of the autophagy gene LC3. Starvation induces the expression of LC3, ATG5, and Beclin-1 in an IKK-dependent manner (Comb et al., 2011).

In addition to the classical NF-κB pathway, the non-classical NF-κB signaling pathway plays a key role in various biological functions, including chronic inflammation and tumorigenesis (Sun, 2017). The activation of non-classical NF-κB signaling is mainly dependent on the abundance of NF-κB family members p100/p52. The transcription factor p100 inhibited the pathway in the resting state. Upon activation of this pathway, p100 was processed as a precursor to transcriptionally active p52 *via* the proteasome pathway, which in turn activated the non-classical NF-κB pathway. p100/p52 was further activated by IKKα activation, and eventually, p52 enterd the nucleus leading to inflammatory activation. The substrate receptor p62 in selective autophagy inhibited the non-classical NF-κB signaling pathway by recognizing the K63 ubiquitinated chain at the N-terminal end of p52/p100, which in turn induced autophagic degradation of p52/p100 (Chen et al., 2020).

A limitation of this study was that there was no evidence of crosstalk between the NF-κB pathway and autophagy in HLD-relieved UC. Further studies should be conducted to explore the roles and mechanisms of non-canonical NF-κB pathway genes such as IKKα and p52/p100 and autophagy in HLD treatment of UC.

## Conclusion

The study findings suggested that HLD protected against DSS-induced colitis in mice. In addition, HLD protected against LPS-induced inflammation in NCM460 cells by inhibiting autophagy and the NF-κB pathway. There was no crosstalk between autophagy and the NF-κB pathway, which may have synergistic anti-inflammatory effects.

## Data Availability

The raw data supporting the conclusion of this article will be made available by the authors, without undue reservation.

## Abbreviations

CRP, C-reactive protein; DAB, diaminobenzidine; DAI, Disease activity index; DSS, Dextran sulfate sodium; ESR, erythrocyte sedimentation rate; HE, hematoxylin-eosin; HLD, Huangkui lianchang decoction; HLD-DS, Drug serum-containing HLD; IBD, Inflammatory bowel disease; IKK, IκB kinase; LPS, Lipopolysaccharide; 3-MA, 3-Methyladenine; NCM, Normal colon mucosa; PDTC, Pyrrolidine dithiocarbamate; PVDF, polyvinylidene fluoride; RAPA, Rapamycin; SD, standard deviation; UC, Ulcerative colitis; UHPLC-MS/MS, ultra-high performance liquid chromatography-tandem mass spectrometry.
